# Does augmentation increase the pull-out force of symphyseal screws? A biomechanical cadaver study

**DOI:** 10.1007/s00068-022-01963-6

**Published:** 2022-04-01

**Authors:** Adrian Cavalcanti Kußmaul, Fanny Schwaabe, Christopher Alexander Becker, Christian Kleber, Christoph Linhart, Christoph Thorwächter, Bianka Rubenbauer, Wolfgang Böcker, Axel Greiner

**Affiliations:** 1 Department of Orthopaedics and Trauma Surgery, Musculoskeletal University Center Munich (MUM), University Hospital, LMU Munich, Marchioninistr. 15, 81377 Munich, Germany; 2 Department of Orthopaedics, Trauma and Plastic Surgery, University Hospital Leipzig, Liebigstraße 20, 04103 Leipzig, Germany

**Keywords:** Biomechanics, Pubic symphysis, Augmentation, Osteosynthesis, Pull-out force

## Abstract

**Purpose:**

Open reduction and internal fixation using anterior plate osteosynthesis currently represents the gold standard for the treatment of symphyseal disruptions. Since postoperative screw loosening with consequent implant failure is frequently observed, this study aims to evaluate if and to what extent augmentation can increase the pull-out force of symphyseal screws to improve the constructs stability.

**Methods:**

Twelve human cadaveric anterior pelvic rings were separated at the symphyseal joint for bilateral testing, consequently achieving comparable sites. First, one non-augmented screw was drilled into the superior pubic ramus, whereas the contralateral side was primarily augmented. The screws were then withdrawn with a constant speed of 10 mm/min and the fixation strengths determined by the force (N) displacement (mm) curve. Finally, the primary non-augmented site was secondary augmented, representing revision surgery after initial implant failure, and the corresponding fixation strength was measured again.

**Results:**

Augmentation compared to non-augmented screws displayed significantly higher pull-out forces with an increase in pull-out force by 377% for primary and 353% for secondary augmentation (*p* < 0.01). There was no significant difference in the pull-out force comparing primary and secondary augmentation (*p* = 0.74).

**Conclusions:**

Primary and secondary augmentation significantly increases the stability of symphyseal screws and, therefore, potentially decreases rates of implant failure.

**Supplementary Information:**

The online version contains supplementary material available at 10.1007/s00068-022-01963-6.

## Introduction

Pelvic ring fractures involving disruption of the symphyseal joint are commonly the result of high impact trauma [1, 2]. In accordance with the AO classification and with regard to concomitant non-displaced posterior pelvic ring injuries, anterior pelvic ring fractures are classified as type 61-B1, 61-B2 or type 61-B3 fractures [3].

Surgical treatment of pelvic ring fractures aims to restore the stability and integrity of the pelvic ring [4]. In case of symphyseal disruption, open reduction and internal fixation using anterior plate osteosynthesis is a well-established treatment method [5, 6].

However, rates of implant failure are high, ranging from 30.9 to 43% [1, 6]. Especially screw loosening, even though not always clinically apparent, is postoperatively commonly observed in 75–81% [7, 8].

In addition, current society faces the challenge of an aging population and consequently of a rise in low-impact pelvic fractures [9–11].

Therefore, compromised bone tissue based on osteopenia and osteoporosis should increasingly be considered a potential stability-limiting factor to surgical implants.

Despite this high and progressively occurring frequency of screw loosening, current literature is lacking sufficient data on potential possibilities to improve the pull-out force of symphyseal screws.

However, numerous biomechanical studies have investigated the influence of augmentation on screws in different fracture entities.

For example, in a study by Sarzier et al., vertebroplasty with Polymethylmethacrylate (PMMA) significantly increased the pull-out force of pedicle screws compared to non-augmented screws in osteoporotic, cadaveric vertebrae [12]. A later published literature review concluded that PMMA allows successful pedicle screw fixation in both osteoporotic specimens and after initial screw failure of non-augmented screws [13].

Another biomechanical study showed an improvement in the primary stability of locked plate constructs at the proximal humerus through augmentation of screws [14].

The concept of stability-enhancing augmentation has also already been tested with promising results for sacroiliac (SI) screws at the posterior pelvic ring [15]. In a biomechanical study by Suero et al., a single augmented screw was able to achieve a comparable stability to two non-augmented screws [15]. In addition, Grechenig et al. revealed a significantly higher pull-out force for augmented SI-screws in contrast to non-augmented screws [16].

Consequently, the aim of this study was to explore the applicability of augmentation for symphyseal implants and to hereby increase the pull-out force of symphyseal screws. Furthermore, we evaluated whether secondary augmentation after previous non-augmented screw failure, imitating a revision surgery setting, could provide comparable stability to primarily augmented screws.

## Materials and methods

After obtaining written consent from the ethics committee (approval no. 210–16) and the relatives of the donors, 12 human anterior pelvic rings were used in this study. The pelvises were collected between 2016 and 2018. Three pelvises were female and nine were male with a mean age of 60 years (age span between 25 and 74 years) and a mean bone mineral density of 122.2 mg Ca–Ha/ml (ranging from 63.2 mg Ca–Ha/ml to 178.8 mg Ca–Ha/ml), measured at the lumbar column between L3 and L5 using a qCT.

**Fig. 1:**
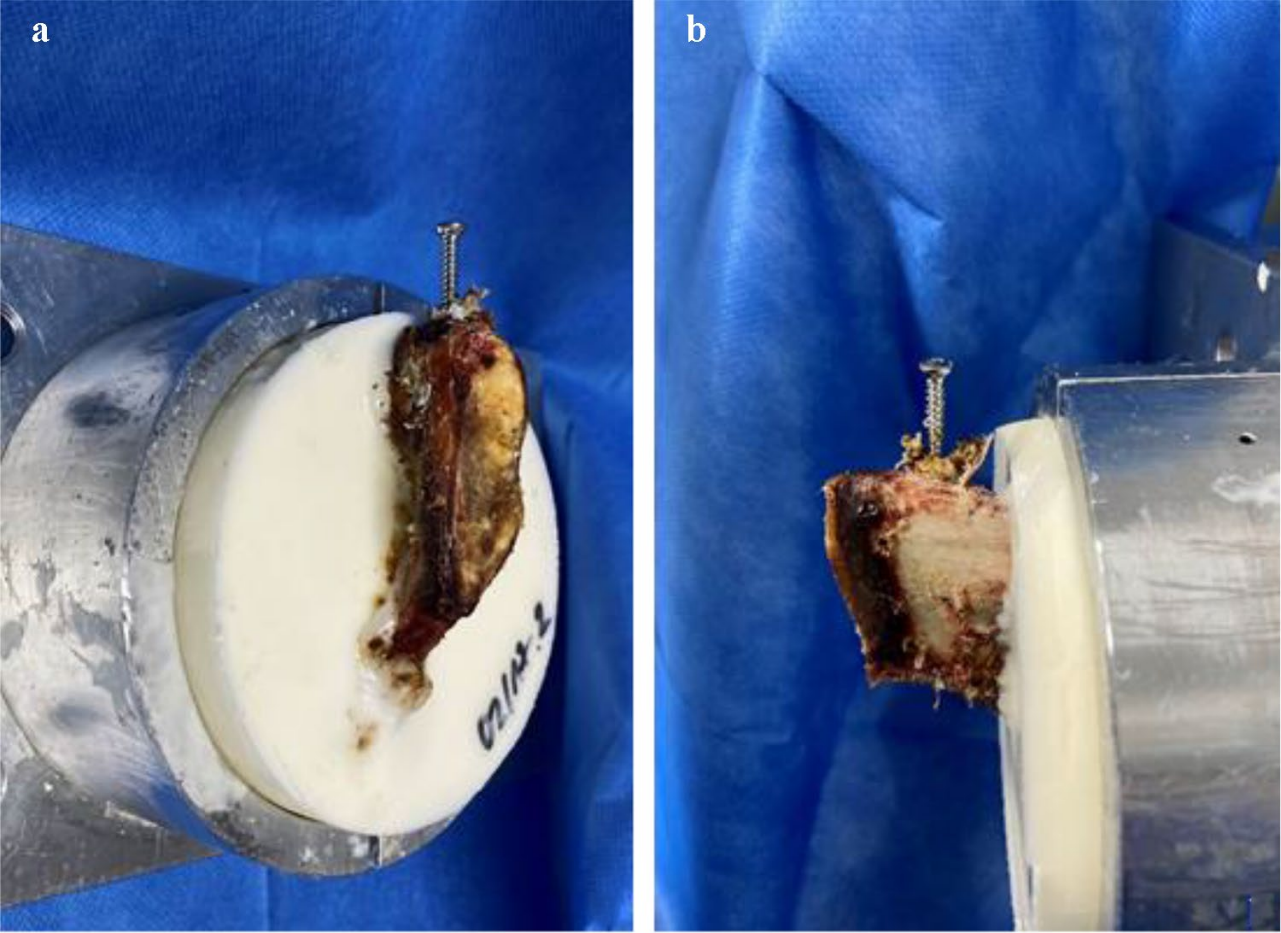
Embedded anterior pelvic ring after pull-out of cemented screws **a** medial view, **b** frontal view

**Fig. 2:**
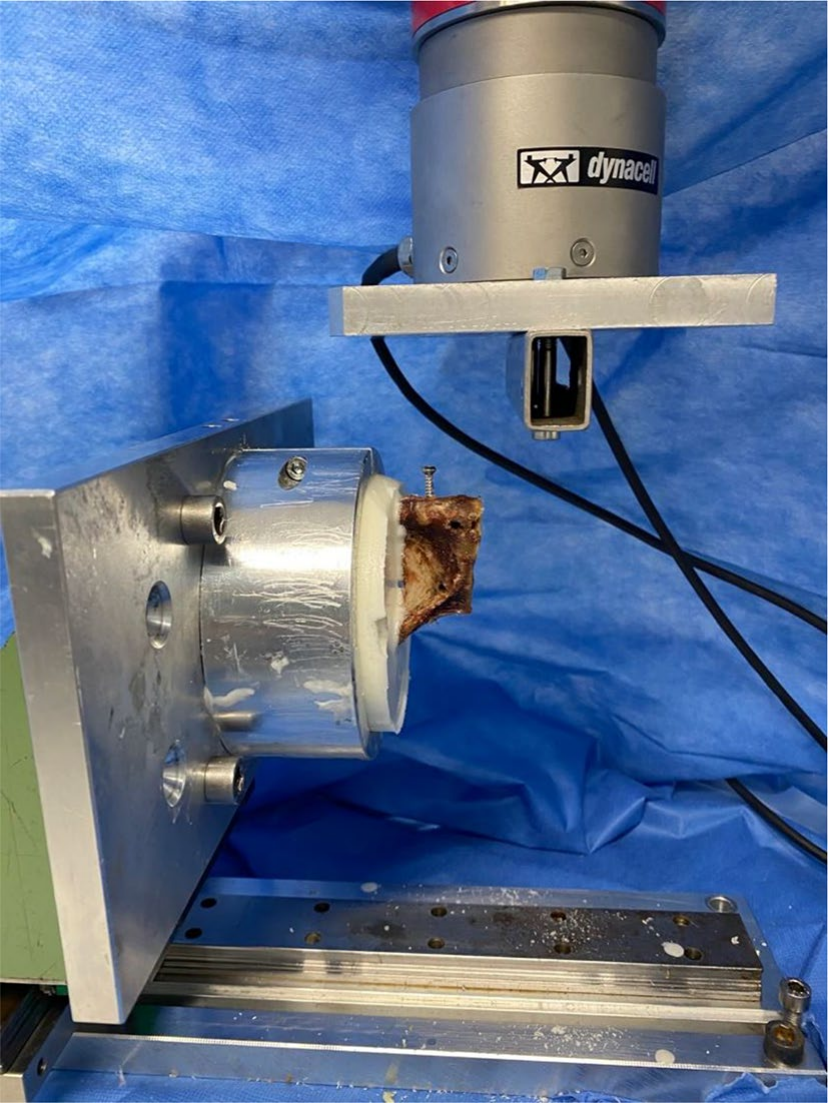
Experimental setup prior to pull-out (linear slide used to eliminate shear forces and to position specimen)

The specimens were thawed 1 day prior to the experiment. For independent testing, the pelvic rings were separated at the symphyseal joint. Then, each partial ring was embedded into cylindrically formed metal containers and attached to the biomechanical testing machine (Instron ElectroPulsTM E10000 Linear-Torsion, Norwood, MA, USA) (see Figs. 1 and 2). Next, a screw (DePuySynthes Cortex Screw 3.5 mm, 50 mm) was inserted 1 cm laterally from the median line into the superior pubic ramus parallel to the symphyseal surface, representing the medial screw in a plate osteosynthesis. The screws were placed monocortically to approach identical screw placement as well as direction and to ultimately achieve comparable results.

Following previous studies, the screw was withdrawn with a constant speed of 10 mm/min [17, 18]. In accordance with other studies, the pull-out strength was defined by the maximum force before a decrease in load was recorded [18–20].

Contemporaneous on the contralateral symphyseal side, 2 ml (range 1.5–2.5 ml) of PMMA were inserted into a pre-drilled bone channel (2.8 mm) after which a symphyseal screw was inserted, representing a primary augmented screw (Cement: Palacos^®^, Heraeus Medical GmbH, Hanau, Germany). After completed polymerization reaction, the screw was then pulled out equivalently as described above and its corresponding pull-out force (N) was measured.

Finally, the primary side, on which the non-augmented screw had been torn out, was secondary augmented as described above and a subsequent pull-out was performed, simulating the conditions after initial non-augmented screw loosening in a surgical revision setting.

**Fig. 3:**
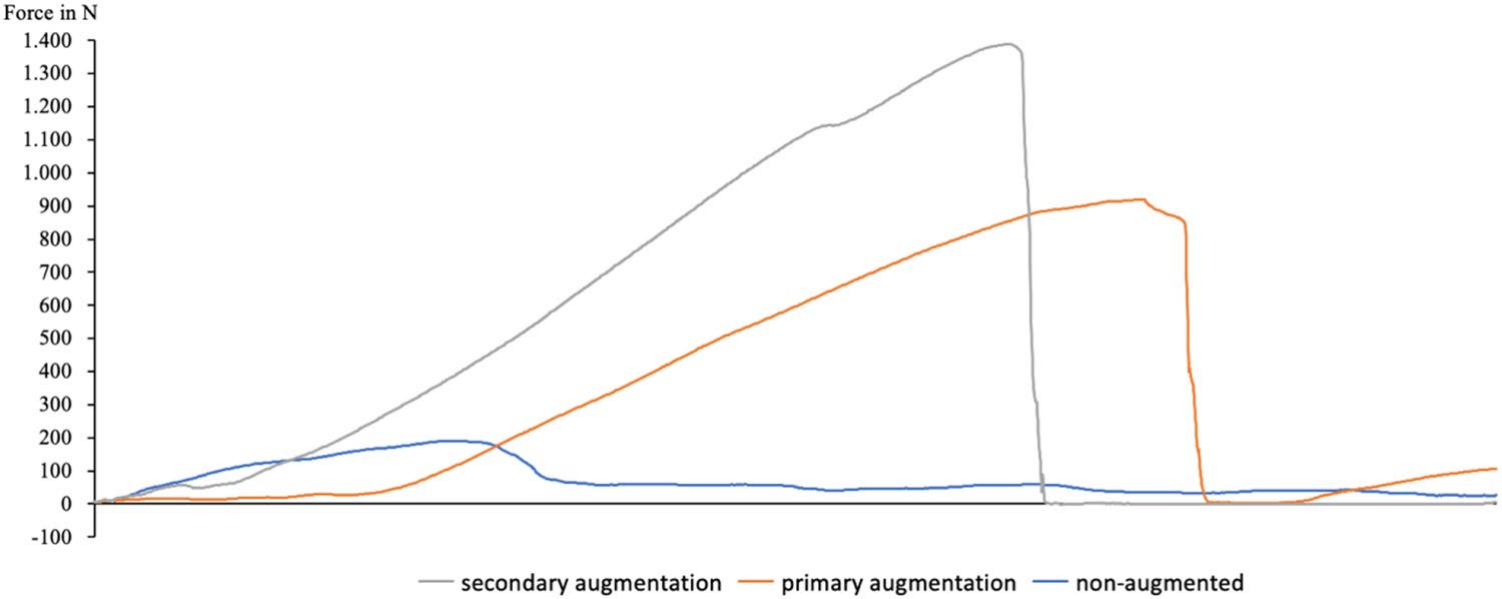
Exemplary pull-out force curve of specimen #7. Indication of graphic programs used for figure creation: MS Office PowerPoint and Efficient Elements Version 3.9.9600.1

**Fig. 4:**
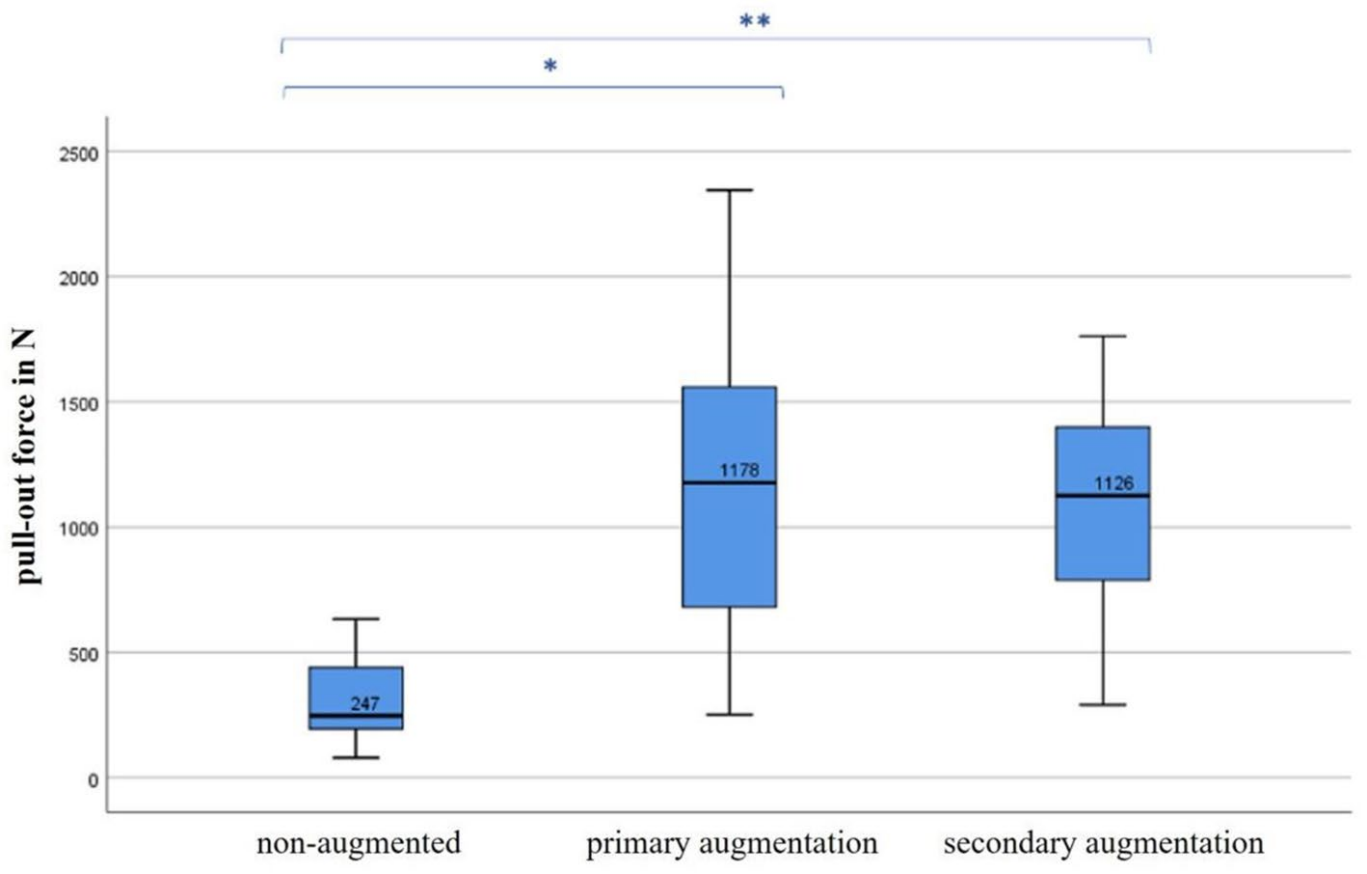
Boxplot of the pull-out-forces regarding the screw fixation technique. Significant difference in both the pull-out forces without and after primary augmentation (*) and those without and after secondary augmentation (**). Indication of graphic programs used for figure creation: IBM SPSS Statistics (Windows, version 26.0, IMB Corp., Armonk, N.Y., USA)

Afterwards, the three different pull-out conditions—non-augmented, primary augmentation and secondary augmentation—were compared in terms of their maximum forces (see Figs. 3 and 4).

For further statistical analysis, the hemi pelvises with the primary non-augmented screws were divided into two groups with an arbitrarily set cutoff value of their pull-out forces: Group 1 included specimens showing a pull-out force below 250 N and group 2 those with values above 250 N. The cutoff value was set to 250 N based on the consequent formation of two groups of equal sample size (*N* = 6), enabling rational statistical testing. In addition, reliability of the group division was verified by a *t* test, which showed that the pull-out forces of the groups differed significantly from each other (*p* = 0.013). Subsequently, it was analysed whether group 1 further maintained a lower pull-out force than group 2 after secondary augmentation.

Statistical analysis was carried out using IBM SPSS Statistics (Windows, version 26.0, IMB Corp., Armonk, N.Y., USA). The Shapiro–Wilk Test and the Kolmogorov–Smirnov Test were used to assess normal distribution of the data. If the variables displayed a normal distribution, a *t* test was used for comparison. Otherwise, a Wilcoxon test was performed. Statistical significance was set to *p* ≤ 0.05.

## Results:

The mean pull-out force of the non-augmented screws was 307.84 ± 185.70 N, while the primarily augmented screws showed a mean pull-out force of 1161.75 ± 597.59 N. The secondarily augmented screws displayed a mean pull-out force of 1087.21 ± 448.97 N (Table 1).

**Table 1:** Tabular display of the pull-out forces in respect to screw fixation technique

	Non-augmented	Primary augmentation	Secondary augmentation
Mean (N)	307.84	1161.75	1087.21
Standard deviation (N)	185.70	597.59	448.97
Median (N)	247.09	1177.65	1126.07
N	12	12	12

Both augmented screws, regardless of their primary or secondary augmentation, showed significantly higher pull-out forces than the non-augmented screws (both *p* < 0.01). There was no significant difference in the pull-out forces considering primary and secondary augmentation (*p* = 0.74) (Fig. 4).

Furthermore, group 1 (initial pull-out force below 250 N, mean pull-out force before secondary augmentation: 176.38 ± 59.13 N, mean pull-out force after secondary augmentation: 984.23 ± 554.02 N) did not display a significantly lower pull-out force after secondary augmentation than group 2 (initial pull-out force above 250 N, mean pull-out force before secondary augmentation: 439.29 ± 175.77 N, mean pull-out force after secondary augmentation: 1190.20 ± 333.29 N) (*p* = 0.45).

## Discussion

After anterior fixation rates of screw loosening with consequent implant failure can occur, even if not always clinical apparent, in up to 81% [7, 8]. Based on the current aging society, osteopenia and osteoporosis further impede the stability of osteosynthesis constructs, especially regarding screw anchorage [9–11, 21].

Although various studies have explored the promising effect of screw augmentation in different fracture entities, for example in the spine [12, 21], the humerus [14], the femoral head [22] or at the posterior pelvic ring [15, 16], there is no data investigating the effect of primary or secondary augmentation in symphyseal screws.

In this study, the results show an almost fourfold greater pull-out force (377%) for primarily augmented symphyseal screws compared to non-augmented symphyseal screws (*p* < 0.01), highlighting the potential capability of primary augmentation to increase the stability of symphyseal plating.

Our findings, therefore, correspond with a study of Suero et al., in which the authors were able to demonstrate comparable biomechanical stability of a single primary augmented sacroiliac screw compared to a non-augmented double-screw technique for the treatment of incomplete pelvic ring fractures [15]. The authors also found that a non-augmented single screw osteosynthesis provided significantly worse stability compared to a single augmented screw technique [15].

Regarding compromised bone quality and the concomitant challenge of screw anchorage, König et al. further examined primarily augmented minimally invasive sacroiliac screws for the treatment of sacral insufficiency fractures and were able demonstrate promising biomechanical results in terms of fracture stabilization and excellent postoperative clinical results based on an instant patient mobilization and immediate pain relief [21].

Another study investigating the effect of primary augmentation on sacroiliac screws in osteoporotic posterior pelvic ring fractures was performed by Oberkircher et al. [23]. In accordance with our results, the authors were able to demonstrate a significant increase in screw stability with augmentation compared to non-augmented sacroiliac screws [23].

In a study examining augmentation on spinal injuries with poor bone quality, Kolb et al. showed an increased biomechanical stability displayed by a significant higher pull-out force regarding screw anchorage when using augmentation. However, the authors also underlined the concomitant change in biomechanical properties, potentially increasing the risk of adjacent fractures in the spine [24].

Primary pedicle screw augmentation in the lumbar spine was also found to increase osteosynthesis’ fixation strength in osteoporotic vertebrae by Burval et al. [25] and Saadeh et al. [26].

Regarding secondary augmentation, our study was further able to demonstrate a 3.5-fold greater pull-out force (353%) for secondarily augmented symphyseal screws compared to non-augmented symphyseal screws (*p* < 0.01), another phenomenon not yet examined in current literature for the pubic symphysis.

However, secondary augmentation has been proven to significantly increase the stability of primarily non-augmented osteosynthesis methods in a revision surgery setting, for example in the lumbar spine [27].

Weiser et al. showed a significant increase in pull-out force and failure load of augmentation after screw loosening for pedicle screws in the lumbar spine [27].

Derincek et al. also investigated the effect of secondary PMMA augmentation in the revision for initial pedicle screw loosening in osteoporotic vertebrae [28]. The authors found that PMMA augmentation is the most promising way to increase the bone–metal interface, especially when compared to the sole replacement of pedicle screws by longer or thicker screws due to here existing risk of neurovascular or pedicle injury [28].

Overall, our study shows excellent biomechanical results regarding the significant superior biomechanical stability of both primary and secondary screw augmentation at the pubic symphysis.

Regarding the direct comparison between primary and secondary augmentation, we did not find a significant difference (*p* = 0.738), emphasizing the promising applicability of both augmentation techniques for the treatment of symphyseal disruptions.

Nevertheless, in case of symphyseal implant failure prior to adequate healing and the eventual need for operative revision, occurring in up to 22% of the cases [8], this study was able to identify secondary augmentation for symphyseal screws as a promising alternative for the revision treatment of symphyseal disruptions after initial implant failure. This prospectively may be a superior option compared to the current revision techniques, which include the subsequent use of longer plates or double plate osteosynthesis with an increase in surgical invasiveness and concomitant higher perioperative complication rates or the application of external fixators, exposing the patient to potential pin infections, reduced mobility and decreased patient comfort [8, 29]. In contrast, a secondary augmentation not only ensures sufficient biomechanical stability but also maintains the prior surgical invasiveness and limits the amount of inserted foreign material.

Considering the biomechanical differentiation of pelvises with a non-augmented pull-out force below 250 N and those above 250 N, this study did not find a significant difference between their pull-out forces after secondary augmentation. This emphasizes the immense increase in stability after secondary augmentation independently from the initial pull-out force.

An optimum clinical fracture management, however, is based on a multifactorial concept, consequently not only including biomechanical aspects but also considering the patients age, their comorbidities and potential compliance. This stresses the importance of this study and the consequent need for further biomechanical and in-vivo studies.

The main limitation to this study is the in vitro character, consequently only allowing a hypothetical transferability of the results to a clinical setting. However, the use of fresh frozen human pelvises without formalin fixation allows the closest approach possible to an in-vivo setting. Furthermore, the axial direction of the pull-out forces does not represent the complex interaction of forces acting on an in-vivo symphyseal plate. Also, monocortical screw placement does not fully represent current clinical standard, which is performed through bicortical screw fixation, nevertheless allows comparable results based on approximately identical screw positioning, insertion length and screw direction.

Yet, the axial forces used in this experiment meet the current standards for the biomechanical evaluation of pull-out forces [16, 17] and allow a standardized reproducibility and exact measurement of the data.

Ultimately, both primary and secondary augmentation significantly increased the pull-out forces of symphyseal screws, independently of initial non-augmented pull-out force. This consequently expands surgical possibilities for both primary treatment and revision surgery after frequently seen implant failure.

## Supplementary Information

Below is the link to the electronic supplementary material.

Supplementary file1 (XLSX 44025 KB)

## Data Availability

All data is available upon request.
